# Effects of dynamic body weight support on functional independence measures in acute ischemic stroke: a retrospective cohort study

**DOI:** 10.1186/s12984-023-01132-9

**Published:** 2023-01-16

**Authors:** Justin Huber, Nicholas Elwert, Elizabeth Salmon Powell, Philip M. Westgate, Emily Hines, Lumy Sawaki

**Affiliations:** 1 Department of Physical Medicine and Rehabilitation, University of Kentucky, Lexington, KY USA; 2 Department of Mechanical Engineering, University of Kentucky, Lexington, KY USA; 3 Department of Biostatistics, University of Kentucky, Lexington, KY USA; 4 College of Medicine, University of Kentucky, Lexington, KY USA; 5 Department of Physical Medicine and Rehabilitation, Mayo Clinic, Rochester, MN USA

**Keywords:** Cerebrovascular accident, Gait, Assistive technology, Robotics, Inpatient rehabilitation

## Abstract

**Background:**

Stroke remains a major public health concern in the United States and a leading cause of long-term disability in adults. Dynamic body weight support (DBWS) systems are popular technology available for use in clinical settings such inpatient rehabilitation. However, there remains limited studies in such inpatient settings that compare DBWS to standard of care (SOC) using real world outcome measures. For survivors of acute ischemic stroke, we determine if incorporating a dynamic body weight support (DBWS) system into inpatient therapy offers greater improvement than standard of care (SOC).

**Methods:**

A retrospective chart review included 52 individuals with an acute ischemic stroke admitted to an inpatient rehabilitation facility. Functional Independence Measure (FIM) data, specifically changes in FIM at discharge, served as the primary outcome measure. Patient cohorts received either therapies per SOC or therapies incorporating DBWS. Regardless of cohort group, all patients underwent therapies for 3 h per day for 5 days a week.

**Results:**

For both groups, a statistically and clinically significant increase in total FIM (P < 0.0001) was observed at discharge compared to at admission. Improvements for the DBWS group were significantly greater than the SOC group as evidenced by higher gains in total FIM (p = 0.04) and this corresponded to a medium effect size (Cohen’s *d* = 0.58). Among FIM subscores, the DBWS group achieved a significant increase in sphincter control while all other subscore changes remained non-significant.

**Conclusions:**

This preliminary evidence supports the benefit of using DBWS during inpatient rehabilitation in individuals who have experienced an acute ischemic stroke. This may be due to the greater intensity and repetitions of tasks allowed by DBWS. These preliminary findings warrant further investigations on the use of DBWS in inpatient settings.

## Introduction

Stroke continues to be a major public health concern in the United States and one of the leading causes of long-term disability in adults with approximately 795,000 new cases every year [1]. Unfortunately, as evidenced by ischemic stroke incidence and stroke mortality, the stroke burden is unfortunately increasing in some regions of the country and particularly areas with socioeconomic and healthcare disparities [2, 3]. While significant advancement in recent medical care has increased survival post-stroke, approximately 5 million Americans are still living with residual deficits with an estimated healthcare cost of $46 billion each year. Stroke-related healthcare costs are projected to reach more than $94 billion per year by 2035 in the United States [1]. Therefore, it is of paramount importance to find new ways to improve rehabilitation outcomes and quality of life for people with stroke.

The concept of neuroplasticity plays a crucial role in rehabilitation outcomes [4]. Remarkable neuroplastic changes in corticospinal systems have been demonstrated during and/or after the intense performance of motor activities [5]. Generally speaking, neuroplastic change occurs more readily as activities become more intensive and repetitive with progressive challenges and salience [6]. Such activities have a greater likelihood of leading to lasting change in functional performance if the activities require active participation, problem-solving, and attention to task. It has also been known for a while now that the potential for functional recovery after stroke is greatest during the first 3–6 months following a stroke [7, 8]. Therefore, inpatient rehabilitation occurs during a pivotal time frame from a neuroplasticity perspective.

However, for patients with considerable motor and balance impairments, it is very difficult even in an inpatient setting to implement therapy repetitions with the aforementioned intensity, salience, and progressive challenge [9]. These patients have a justified fear of falling. Moreover, the fall risk is difficult to manage solely with therapy personnel as the current rehabilitation setting in the United States features an already high patient-therapist ratio [10]. Conceivably, the fall risk and the fear of falling are detrimental for neuroplasticity: (a) the attention of the patient and therapist can be more focused on safety measures and distracted from the motor task itself; (b) therapists may select tasks that are safer in lieu of challenging tasks that are more conducive to neuroplastic changes; and (c) repetition goals may be too conservative due to concerns of patient fatigue and the increased fall risk associated with fatigue [11]. These conservative repetition goals in current standards of care are evidenced by a recent study reporting very limited daily step counts for patients undergoing inpatient stroke rehabilitation [12].

**Fig. 1:**
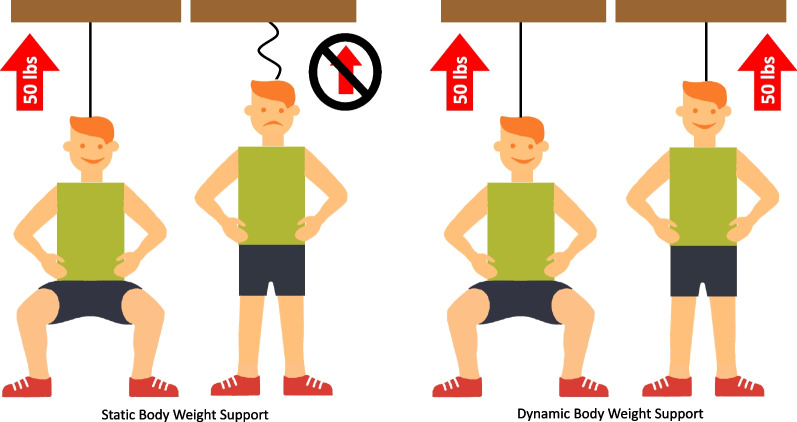
Conceptual illustration of a dynamic body weight support system compared to a static body weight support system

Dynamic body weight support (DBWS) systems are a popular set of technologies that facilitate over-ground therapy and are designed to unload body weight more consistently during dynamic conditions such as movement-based therapy (Fig. 1). This consistent unloading is achieved by means of a sensor, an actuator, and an onboard computer controller. The controller is crucial to creating a feedback control loop, which constantly compares measured load versus the desired load and adjusts rope tension accordingly by means of the actuator. The onboard computer controller inherent to these systems allows novel safety features to reduce fall risk—automatic fall detection and an injury prevention mode. In addition, these systems allow real-time feedback for participants owing to the visual displays of real-time sensor data. By facilitating safe therapy and real-time feedback, DBWS has the potential to foster principles of neuroplasticity. That is, participants may better focus their attention and they may be more motivated for therapeutic activities. Our group has reported beneficial effects of DBWS on inpatient discharge outcomes compared to standard of care in patient populations such as traumatic brain injury and spinal cord injuries [13, 14]. However, it is not known if DBWS can also lead to greater functional recovery during inpatient rehabilitation in a population with an acute ischemic stroke, which is the motivation for this study. In the present study, we evaluate whether over-ground gait and balance training incorporating DBWS leads to greater functional recovery after an acute ischemic stroke compared to standard of care (SOC) as assessed by the Functional Independence Measure (FIM).

## Methods

In this retrospective cohort study, data was collected through chart review of inpatient stroke admissions at a freestanding rehabilitation hospital with a formal partnership with the University of Kentucky. All procedures were in accordance with the Declaration of Helsinki and reviewed by the University of Kentucky Institutional Review Board, which issued a waiver of informed consent. Retrospective patient data was extracted from January 2016 to March 2018 including patient age, admission diagnosis, neuroimaging, length of stay, and use/non-use of DBWS. The inclusion criterion was first acute ischemic stroke with resulting motor deficits significant enough to require admission to the inpatient rehabilitation facility (IRF). Exclusion criteria consisted of multiple strokes, hemorrhagic stroke, no evidence of stroke on neuroimaging, admission to inpatient rehabilitation for reasons other than stroke, and very short/long lengths of stay (LOS). A very short LOS was considered less than 10-days as supported by large studies of “short stay transfer rates” among Medicare beneficiaries [15], and a very long LOS was considered greater than 35 days, which represents three standard deviations above average LOS per very large studies of Medicare beneficiaries [16]. Upon availability of the DBWS, therapy staff considered all patients for the DBWS system based on patients’ safety and patients’ ability to use the device during the usual time constraints of inpatient therapy sessions. All patients assigned to the DBWS group were required to have used the DBWS system (ZeroG v3, Aretech, LLC, Ashburn, VA) in at least 2 physical therapy sessions to ensure that they had adequate time to become familiarized with the system. Therapy using the DBWS system was delivered by therapists properly trained in the use of the device. For the control group, historical medical records were provided in large batches from the IRF facility for patients meeting inclusion criteria. Among these patient records, those with admission and discharge dates nearest the timeframe of the experimental group were prioritized.

A total of 52 patients was included for study analysis (26 in the DBWS group and 26 in the SOC group). All the participants who met the inclusion criteria during the study period were included in the study analysis. That is, the group sizes were not calculated a priori. For both cohort groups, all patients received 3 h of therapy per day, 5 days per week as required by the Centers for Medicare and Medicaid Services [17, 18]. Therapy was comprised of hour-long sessions in at least 2 of the following domains: physical therapy, occupational therapy, and/or speech therapy. Activities performed by the patients in both the DBWS and SOC groups were determined by each individual’s physical therapist. Regarding the DBWS group, the patients receiving this intervention were assigned to physical therapists who had received specialized training on the DBWS system. Patients in the DBWS group did not receive additional time for therapy; rather, DBWS was incorporated into the standard 3 h of therapy per day.

The primary outcome measure of this study was based on the FIM instrument, which was performed at admission and discharge for all inpatients at the rehabilitation hospital. This instrument is an observational assessment that consists of 18 items within physical and cognitive function domains, and it is validated for use in stroke populations [19–21]. Each item is scored according to an ordinal scale that reflects level of dependence, with 1 representing complete dependence and 7 representing complete independence, with total scores ranging from 18 to 126. In an acute stroke population, the minimal clinically important difference (MCID) is 22 points for total FIM [22].

Statistical analysis was performed using SPSS 28 (IBM, Armonk, NY). Results were considered statistically significant when p < 0.05 based on two-tailed tests. Patient data was grouped by whether an individual did (DBWS) or did not (SOC) use the DBWS system while inpatient. Groups were assessed for normality using the Shapiro-Wilks test and for homogeneity of variance using Levene’s test. Parametric tests were applied to normally distributed data, and non-parametric tests were applied otherwise. Between-group differences for age at baseline, total FIM at baseline, length of stay (LOS), and total FIM gain were assessed with a two-sided, independent samples t-test. Within-group comparison of total FIM at admission versus discharge was evaluated with two-sided, paired samples t-tests. Of primary interest was the between-group difference in total FIM gain. To help understand results with respect to total FIM gain, a post-hoc analysis utilized Wilcoxon signed rank tests and Mann Whitney U tests to evaluate baseline and change in FIM subscores. Given the preliminary nature of this retrospective study, a correction for multiple comparisons was not applied during statistical analyses.

## Results

For a total of 52 individuals with acute ischemic stroke requiring IRF admission, data was successfully extracted from the medical record and subsequently analyzed. The cohort receiving DBWS included 26 individuals, and the cohort receiving SOC included 26 individuals. Regarding group characteristics, variance was homogeneous and distributions were normal with exception of FIM subscores. There were no statistically significant differences between the cohort groups (Table 1).

**Table 1:** Group characteristics

	DBWS	SOC	DBWS—SOC
	Mean (SEM), (Range)		Mean (SEM), (p-value)
Age (years)	66.7 (2.8) (45–89)	71.0 (2.4) (48–90)	− 4.3 (3.7) (0.244)^a^
LOS (days)	20.7 (1.0) (12–31)	19.1 (0.9) (10–28)	1.7 (1.4) (0.231)^a^
Baseline Total FIM	45.7 (2.7) (23–73)	42.5 (2.9) (20–69)	3.3 (4.0) (0.415)^a^
Baseline FIM Locomotion	2.6 (0.3)	3.0 (0.4)	– (0.106)^b^
Baseline FIM Mobility	5.2 (0.5)	4.7 (0.5)	– (0.308)^b^

*DBWS* dynamic bodyweight support, *SOC* standard of care, *LOS* length of stay, *FIM* functional independence measure, *SEM* standard error of the mean

^a^Between groups comparison using two-sided, independent samples t-test

^b^Between groups comparison using Mann Whitney U test given non-normal distributions

**Fig. 2:**
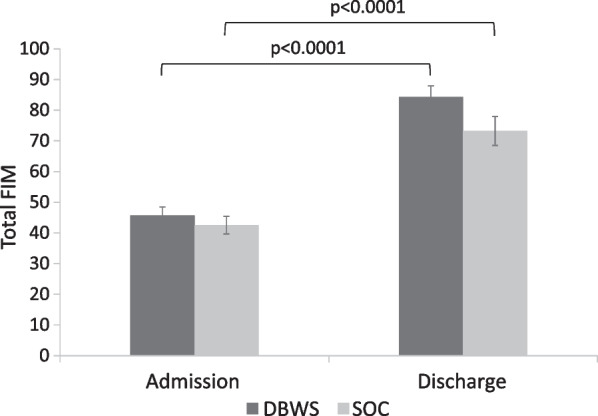
Significant increases from admission to discharge were observed for both groups in Total FIM score. Error bars indicate standard error of the mean. *FIM* functional independence measure, *DBWS* dynamic body weight support, *SOC* standard of care

**Fig. 3:**
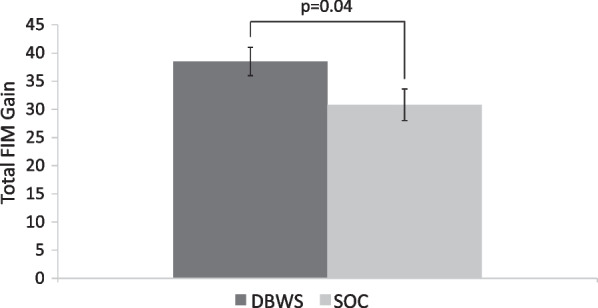
Patients who received therapy incorporating DBWS were found to have significantly greater gains in Total FIM compared to those who received SOC. Error bars indicate standard error of the mean. *FIM* functional independence measure, *DBWS* dynamic body weight support, *SOC* standard of care

Within-group analysis comparing admission versus discharge total FIM revealed significant score increases (p < 0.0001) for both groups (Fig. 2). Both DBWS and SOC within-group changes exceeded MCID for total FIM indicating clinically significant improvements for both groups. A between-group comparison showed significantly greater gains in total FIM for the DBWS group versus the SOC group with an estimated difference in mean gain of 7.8 [95% CI: 0.3, 15.3; p = 0.04], which corresponds to a medium effect size (Cohen’s *d* = 0.58) (Fig. 3).

In a post-hoc analysis of FIM subscores, within-group comparisons revealed significant increases in all subscores for both groups (Table 2). A between-group comparison showed a significantly greater gain in the sphincter control subscore for the DBWS group versus the SOC group [p = 0.03]. While improvements in the other median subscores were observed, none of these subscore changes reached statistical significance.

**Table 2:** FIM subscore comparisons

FIM Subscore^a^	DBWS				SOC			DBWS—SOC
	Median	IQR	P-value^b^		Median	IQR	P-value^b^	P-value^c^
Total Motor	31	(20,41)	< 0.0001		27.5	(17,32)	< 0.0001	0.07
Mobility	7.5	(4,10)	< 0.0001		6	(2,9)	< 0.0001	0.31
Locomotion	5.5	(4,7)	< 0.0001		5	(2,6)	< 0.0001	0.11
Self Care	14.5	(10,18)	< 0.0001		12.5	(7,16)	< 0.0001	0.16
Sphincter	5.5	(2,9)	< 0.0001		2	(0,5)	< 0.0001	0.03
Total Cognitive	7	(3,8)	< 0.0001		6	(4,8)	< 0.0001	0.51
Cognition	3	(2,4)	< 0.0001		3	(2,4)	< 0.0001	0.80
Social Cognition	4	(2,5)	< 0.0001		3	(2,5)	< 0.0001	0.46

*FIM* functional independence measure, *DBWS* dynamic bodyweight support, *SOC* standard of care, *IQR* interquartile range

^a^Mobility includes bed-to-chair/toilet/shower transfer; Locomotion includes ambulatory/wheelchair and stairs; Self Care includes eating, grooming, bathing, upper/lower body dressing, and toileting; Sphincter includes bladder/bowel management; Cognition includes problem solving and memory; Social Cognition includes cognitive comprehension, expression, and social interaction

^b^P-values from Wilcoxon signed rank test for within-group comparison discharge versus admission scores

^c^P-values from a Mann Whitney U Test comparing score changes between groups

## Discussion

Based on this retrospective cohort study, therapy utilizing DBWS during inpatient rehabilitation yields larger gains in function compared to SOC in patients with acute ischemic stroke. Patients whose therapy incorporated the DBWS demonstrated a significantly higher improvement in total FIM at completion of inpatient rehabilitation. Additionally, patients using the DBWS achieved significant improvements in bowel and bladder function based on FIM subscores.

Previous studies have supported the functional benefits of high-dose gait training in stroke populations, but its implementation is not without barriers. Klassen et al. performed a prospective and randomized study to evaluate the dose-relationship of walking exercises during inpatient rehabilitation after stroke [23]. They concluded that higher doses of gait training can yield greater functional outcomes, greater endurance, and higher quality of life compared to SOC and to lower dose of training. While the study by Klassen et al. demonstrated crucial data to optimize training protocols during inpatient rehabilitation, all participants included in their study were able to ambulate at least 5 m with the assistance of a maximum of one person. This requirement may exclude a substantial number of patients as previous literature suggests up to 25% of stroke survivors are unable to walk without full assistance [24]. In order to implement higher doses of gait training for a wider spectrum of stroke survivors during inpatient rehabilitation, it may be necessary to utilize technologies. This is particularly relevant considering regions of the country that face high patient-therapist ratios and patient populations with increased comorbidities [25, 26].

Various technologies are available to overcome barriers to higher training doses. Robotic rehabilitation devices which were designed with clinical applications in mind have been shown to maintain patient safety while allowing activity programs with sufficient intensity and repetitions [27, 28]. However, there is a lack of clinical evidence demonstrating that these robotic devices can in fact augment functional recovery particularly in inpatient rehabilitation settings. Furthermore, in spinal cord injury research, a large systematic review applying robotic rehabilitation together with treadmill training did not show superior outcomes compared to over-ground therapies [29].

DBWS systems, among a newer set of technologies, offer several features that may be important to higher doses of gait training and of other task-based activities during post-stroke rehabilitation. Firstly, they allow for static and dynamic weight unloading with remarkably natural ground-reaction forces during a therapy intervention [30]. This is important because aberrancies in afferent feedback may contribute to less functional patterns of leg muscle activation during human locomotion [31]. DBWS also offers an advantage over treadmill training with BWS because it allows safe performance of a variety of functionally relevant activities. These include transfers, over-ground gait, activities of daily living, ascending or descending stairs, and balance tasks. That is, salience across a number of tasks is achievable with DBWS systems versus treadmill-based systems, and this salience can be achieved without jeopardizing participant safety and without overburdening a therapist team.

The DBWS incorporated in this study may also benefit functional recovery through real-time feedback. Feedback has been previously recognized for its importance to neuroplasticity [32]. In recent studies using static BWS methods, a visual feedback feature has been associated with improved functional recovery after stroke [33]. The DBWS technology in the present study includes a fully instrumented robotic trolley with sensors capable of detecting participant movements, both side to side and up and down. The device software can then manipulate this sensor data into real-time visual feedback that the patient can use to understand trends in their performance. This visualization may then benefit the process of motor learning. Learning to perform a motor task entails processes of encoding motor memory, including both explicit and implicit motor learning [34]. Visual feedback from the DBWS may support one or both of these motor learning processes.

With regards to bowel and bladder function, prior literature has suggested the potential of DBWS to improve outcomes in individuals with non-traumatic spinal cord injury [14]. For spinal cord injury, the link between DBWS and sphincter control may be attributable to shared neural pathways, which has been supported in animal studies [35]. Thus, interventions targeting locomotion may be beneficial to bowel and bladder function. Indeed, human studies have shown locomotor training may play such a role [36]. The concept of shared neural pathways may also extend to the level of the brain, and previous functional MRI study has shown the extensive cortical representation shared between locomotion and pelvic floor activity [37]. Interestingly, in studies of healthy adults, regular physical activity contributes to better pelvic floor function [38], and conceivably stroke survivors may benefit similarly with increased activity of locomotion training. The potential benefits between locomotion and sphincter control may, however, be dependent on characteristics of the stroke. For example, prior literature has suggested a potential dependence of sphincter outcomes on the laterality of stroke, which was not accounted for in the current study and should be considered in the future [39]. The evidence from our study again suggests a potential benefit of DBWS on bowel and bladder function, and future studies on this would be worthwhile.

There were no reports of adverse events related to the DBWS technology used in this study. The absence of adverse events may be attributable to the safety of the DBWS during over-ground task-oriented therapy performed by patients. The implications of this include improved patient-perceived safety, reduced patient fear of falling, improved patient attention to task, and minimized disruptions to motor learning.

Of note, the DBWS system in this study seemed well accepted by both the therapy personnel and by the participants—an observation supported by the cohort of patients that willingly used this system multiple times during their IRF hospitalizations. The short duration and inpatient setting of this study limits discussion about the outpatient utility of DBWS and the long-term acceptance of this technology among providers and patients. Long-term acceptance is crucial for successful deployment of new technologies. Literature suggests an alarming rate of abandonment of technologies, which is especially concerning when technology grows more complex and more expensive [40–43].

In summary, our preliminary results indicate the potential of the DBWS systems to promote greater functional improvement in patients with ischemic stroke. This potential might be explained by higher-dose training, the real-time feedback, and/or the reduced fear of falling enabled by this system, which are conducive to the principles of neuroplasticity. Furthermore, the potential of this technology could be realized without increasing burden on a limited therapy workforce. Future longitudinal studies of interest should explore the impact of DBWS on a variety of outcomes including: (a) return to community ambulation; (b) reduction of secondary complications related to modifiable cardiovascular risk factors and/or immobility; (c) increase in balance, patient-perceived safety, and decrease of falls; (d) reduction of spasticity and related medications; (e) enhancement of general quality of life, including decrease in depression; and f) reduction of caregiver burden.

### Study limitations

While our preliminary data suggests evidence of greater functional recovery compared to the control group, several notable limitations are identified. For instance, one limitation was the lack of a standardized training protocol (including length of stay, number of sessions, and intensity). Because data was collected during inpatient rehabilitation, the training protocol was dictated by the regulatory and insurance requirements of an inpatient rehabilitation facility. A lack of standardized dosage of the DBWS system was also a limitation. In our retrospective study, the initiation of and dosing of DBWS was at the discretion of the patient’s therapy team. While the principle of inpatient rehabilitation was kept the same as much as possible considering time constraints of therapy sessions (typically 60-min) and accounting for patient factors (e.g. safety, device acceptance, fatigue), there is opportunity for further standardizing a dosing protocol based on identified “active ingredients” (e.g. step counts) [44]. Furthermore, the retrospective study examined a relatively small number of data elements from the patient chart, and future studies may benefit from further data extraction including time since stroke, patient demographics beyond age, and interruptions to rehabilitation. Additionally, as the present study focused on the acute stroke population, the relevance of our initial findings to subacute and chronic stroke survivors is uncertain. We encourage future prospective, randomized controlled studies that account for factors such as stroke chronicity, patient comorbidities, and rehabilitation setting. Additionally, optimal DBWS parameters, dose–response relationships of this novel intervention, long-term acceptance of this technology, and the implications of this technology on rehabilitation economics and human resources should also be explored in the future.

## Conclusion

Our retrospective cohort study suggests that DBWS can enhance functional recovery during inpatient rehabilitation following ischemic stroke compared to SOC. These findings are considered preliminary and warrant further study in prospective clinical trials.

## Data Availability

The datasets generated and analyzed during the current study are available from the corresponding author on reasonable request.

## Abbreviations

DBWS: Dynamic body weight support
SOC: Standard of care
FIM: Functional independence measure
IRF: Inpatient rehabilitation facility
LOS: Length of stay
MCID: Minimum clinically important difference
SEM: Standard error of the mean
